# Supplementary material for: Dynamic Nucleosome Movement Provides Structural Information of Topological Chromatin Domains in Living Human Cells
Source: PLoS Comput Biol. 2016 Oct 20;12(10):e1005136. doi: 10.1371/journal.pcbi.1005136 (PMC5072619; doi:10.1371/journal.pcbi.1005136)
Supplement: S1 Text — (PDF) [file pcbi.1005136.s001.pdf]

S1 Text

I. DERIVATIONS OF THE THEORETICAL RESULTS

Here we give the complete derivations of the theoretical results. The following calculations are based on standard textbooks related to polymer dynamics [S1] and statistical physics [S2]. The mean-squared displacement (MSD) of the Rouse polymer in the viscoelastic environment was first analyzed by Weber *et al.* [S3], whose work is a useful reference for the flow in the following calculations.

A. The fluctuation-dissipation relation between $\boldsymbol{g}_p(t)$ and $\gamma(t)$

According to the fluctuation-dissipation relation (FDR) for $\boldsymbol{g}(n,t)$,

$$\langle g_\kappa(n,t) g_\lambda(m,t')\rangle = k_\mathrm{B}T\gamma(t-t')\delta(n-m)\delta_{\kappa\lambda}, \tag{S1}$$

the following calculations can be made:

$$\begin{aligned}
\langle g_{p\kappa}(t) g_{q\lambda}(t')\rangle &= \frac{1}{N^2}\int_0^N dn \int_0^N dm \cos\left(\frac{p\pi n}{N}\right)\cos\left(\frac{q\pi m}{N}\right)\langle g_\kappa(n,t) g_\lambda(m,t')\rangle, \\
&= \frac{1}{N^2}\int_0^N dn \int_0^N dm \cos\left(\frac{p\pi n}{N}\right)\cos\left(\frac{q\pi m}{N}\right) k_\mathrm{B}T\gamma(t-t')\delta(n-m)\delta_{\kappa\lambda}, \\
&= \frac{k_\mathrm{B}T}{N}\gamma(t-t')\delta_{\kappa\lambda}\frac{1}{N}\int_0^N dn \cos\left(\frac{p\pi n}{N}\right)\cos\left(\frac{q\pi n}{N}\right), \\
&= \frac{k_\mathrm{B}T}{N}\gamma(t-t')\delta_{\kappa\lambda}\frac{1}{2N}\int_0^N dn \left[\cos\left(\frac{(p-q)\pi n}{N}\right) + \cos\left(\frac{(p+q)\pi n}{N}\right)\right], \\
&= \frac{k_\mathrm{B}T}{N}\gamma(t-t')\delta_{\kappa\lambda}\frac{\delta_{pq}(1+\delta_{p0})}{2}.
\end{aligned} \tag{S2}$$

B. The parameter k_p relates to the variance of $\boldsymbol{X}_p$

At thermal equilibrium via the preaveraging approximation, the memory effect of the friction coefficient vanishes, *i.e.*, $\gamma(t-t') \to 2\bar{\gamma}\cdot\delta(t-t')$. Then, Eq. 5 for $p \geq 1$ can be written as

$$\bar{\gamma}\dot{\boldsymbol{X}}_p(t) = -k_p\boldsymbol{X}_p(t) + \bar{\boldsymbol{g}}_p(t), \tag{S3}$$

where

$$\langle \bar{g}_{p\kappa}(t)\bar{g}_{q\lambda}(t')\rangle = \frac{\bar{\gamma}k_\mathrm{B}T}{N}\delta(t-t')\delta_{\kappa\lambda}\delta_{pq}. \tag{S4}$$

Since this Langevin equation for one degree of freedom corresponds to the Ornstein-Uhlenbeck process described by the stochastic differential equation [S4],

$$dX_{p\kappa}(t) = -\frac{k_p}{\bar{\gamma}}X_{p\kappa}(t)dt + \sqrt{\frac{k_\mathrm{B}T}{N\bar{\gamma}}}\,dB_t, \tag{S5}$$

the variance of $\boldsymbol{X}_p$ becomes

$$\left\langle X_{p\kappa}^2\right\rangle_\mathrm{CD} = \frac{k_\mathrm{B}T/(N\bar{\gamma})}{2k_p/\bar{\gamma}} = \frac{k_\mathrm{B}T}{2Nk_p}, \tag{S6}$$

where $\langle\cdot\rangle_\mathrm{CD}$ represents the average for all nucleosome beads within the chromatin domain (CD) at thermal equilibrium. Thus, this relation implies that the normal-coordinate amplitude satisfies the equipartition theorem at thermal equilibrium.

C. Asymptotic form of $\langle \boldsymbol{X}_p^2 \rangle_{\rm CD}$

Here, we omit the argument t to calculate the thermal average. Using integration by parts, the normal coordinates $\boldsymbol{X}_p \equiv \frac{1}{N}\int_0^N \cos\left(\frac{p\pi n}{N}\right) \boldsymbol{R}(n)\, dn$ are rewritten as

$$\boldsymbol{X}_p = -\frac{1}{p\pi}\int_0^N dn\, \sin\left(\frac{p\pi n}{N}\right) \frac{\partial \boldsymbol{R}(n)}{\partial n}. \tag{S7}$$

Thus, $\langle \boldsymbol{X}_p^2 \rangle_{\rm CD}$ is written as

$$\left\langle \boldsymbol{X}_p^2 \right\rangle_{\rm CD} = \frac{1}{p^2\pi^2}\int_0^N dn \int_0^N dm\, \sin\left(\frac{p\pi n}{N}\right) \sin\left(\frac{p\pi m}{N}\right) \left\langle \frac{\partial \boldsymbol{R}(n)}{\partial n} \cdot \frac{\partial \boldsymbol{R}(m)}{\partial m} \right\rangle_{\rm CD}. \tag{S8}$$

Using

$$\frac{\partial \boldsymbol{R}(n)}{\partial n} \cdot \frac{\partial \boldsymbol{R}(m)}{\partial m} = -\frac{1}{2}\frac{\partial^2}{\partial n\, \partial m}[\boldsymbol{R}(n) - \boldsymbol{R}(m)]^2, \tag{S9}$$

we can rewrite $\langle \boldsymbol{X}_p^2 \rangle_{\rm CD}$ as

$$\left\langle \boldsymbol{X}_p^2 \right\rangle_{\rm CD} = -\frac{1}{2p^2\pi^2}\int_0^N dn \int_0^N dm\, \sin\left(\frac{p\pi n}{N}\right) \sin\left(\frac{p\pi m}{N}\right) \frac{\partial^2}{\partial n\, \partial m} \left\langle [\boldsymbol{R}(n) - \boldsymbol{R}(m)]^2 \right\rangle_{\rm CD}. \tag{S10}$$

Introducing a new variable $l = m - n$ and substituting the size scaling (Eq. 4), we can make the following calculation:

$$\begin{aligned} \left\langle \boldsymbol{X}_p^2 \right\rangle_{\rm CD} &= \frac{1}{2p^2\pi^2}\int_0^N dn \int_{-n}^{N-n} dl\, \sin\left(\frac{p\pi n}{N}\right) \sin\left(\frac{p\pi (l+n)}{N}\right) \frac{\partial^2}{\partial l^2}\left(b_{\rm eff}^2 |n-m|^{2/d_{\rm f}}\right), \\ &= \frac{b_{\rm eff}^2}{2p^2\pi^2}\frac{2}{d_{\rm f}}\left(\frac{2}{d_{\rm f}} - 1\right)\int_0^N dn \left[\sin\left(\frac{p\pi n}{N}\right)\cos\left(\frac{p\pi n}{N}\right) \underline{\int_{-n}^{N-n} dl\, \sin\left(\frac{p\pi l}{N}\right) |l|^{2/d_{\rm f}-2}} \right. \\ &\qquad \left. + \sin\left(\frac{p\pi n}{N}\right)\sin\left(\frac{p\pi n}{N}\right) \underline{\int_{-n}^{N-n} dl\, \cos\left(\frac{p\pi l}{N}\right) |l|^{2/d_{\rm f}-2}}\right]. \end{aligned} \tag{S11}$$

The underlined integrals converge quickly to the following values if p is large:

$$\int_{-n}^{N-n} dl\, \sin\left(\frac{p\pi l}{N}\right) |l|^{2/d_{\rm f}-2} \simeq \int_{-\infty}^{\infty} dl\, \sin\left(\frac{p\pi l}{N}\right) |l|^{2/d_{\rm f}-2} = 0 \tag{S12}$$

and

$$\int_{-n}^{N-n} dl\, \cos\left(\frac{p\pi l}{N}\right) |l|^{2/d_{\rm f}-2} \simeq \int_{-\infty}^{\infty} dl\, \cos\left(\frac{p\pi l}{N}\right) |l|^{2/d_{\rm f}-2}. \tag{S13}$$

Therefore, we can obtain

$$\left\langle \boldsymbol{X}_p^2 \right\rangle_{\rm CD} \simeq \frac{b_{\rm eff}^2}{2p^2\pi^2}\frac{2}{d_{\rm f}}\left(\frac{2}{d_{\rm f}} - 1\right)\frac{N}{2}\, 2\int_0^\infty dl\, \cos\left(\frac{p\pi l}{N}\right) l^{2/d_{\rm f}-2}. \tag{S14}$$

Using the formulas

$$\int_0^\infty \cos(ax)\, x^{b-1}\, dx = \Gamma(b)\cos\left(\frac{\pi b}{2}\right) a^{-b} \qquad \text{and} \qquad z\,\Gamma(z) = \Gamma(z+1), \tag{S15}$$

we can make the following formal calculations:

$$\begin{aligned}
\langle \boldsymbol{X}_p^2 \rangle_{\rm CD} &\simeq \frac{b_{\rm eff}^2 N}{2p^2\pi^2}\,\frac{2}{d_{\rm f}}\left(\frac{2}{d_{\rm f}}-1\right)\Gamma(2/d_{\rm f}-1)\cos\left[\frac{\pi}{2}(2/d_{\rm f}-1)\right]\left(\frac{p\pi}{N}\right)^{1-2/d_{\rm f}}, \\
&= \frac{b_{\rm eff}^2 N^{2/d_{\rm f}}}{2}\,\frac{2}{d_{\rm f}}\,\Gamma(2/d_{\rm f})\cos\left(\frac{\pi}{d_{\rm f}}-\frac{\pi}{2}\right)(p\pi)^{-1-2/d_{\rm f}}, \\
&= \frac{\langle R^2\rangle_{\rm CD}}{2}\,\frac{\Gamma(1+2/d_{\rm f})\,\sin(\pi/d_{\rm f})}{\pi^{1+2/d_{\rm f}}}\,p^{-1-2/d_{\rm f}}, \\
&= \frac{\langle R^2\rangle_{\rm CD}}{2A_{d_{\rm f}}}\,p^{-1-2/d_{\rm f}}, \qquad \text{(S16)}
\end{aligned}$$

where

$$A_{d_{\rm f}} = \frac{\pi^{1+2/d_{\rm f}}}{\Gamma(1+2/d_{\rm f})\,\sin(\pi/d_{\rm f})} \qquad \text{(S17)}$$

is a dimensionless constant depending on the fractal dimension $d_{\rm f}$.

D. The solution of Eq. 9

Performing the Laplace transform to Eq. 9, we obtain

$$\tilde{\gamma}(s)\left[s\,\tilde{C}_p(s) - C_p(0)\right] = -k_p\tilde{C}_p(s), \qquad \text{(S18)}$$

where $\tilde{\gamma}(s)$ and $\tilde{C}_p(s)$ are the Laplace transforms of the functions $\gamma(t)$ and $C_p(t)$, respectively. Since $\gamma(t)$ is defined by Eq. 2, $\tilde{\gamma}(s)$ is derived as follows:

$$\begin{aligned}
\tilde{\gamma}(s) &= \frac{\gamma_\alpha}{\Gamma(1-\alpha)}\int_0^\infty e^{-st}t^{-\alpha}\,dt, \\
&= \frac{\gamma_\alpha}{\Gamma(1-\alpha)}s^{\alpha-1}\int_0^\infty e^{-y}y^{(1-\alpha)-1}\,dy, \\
&= \gamma_\alpha s^{\alpha-1}. \qquad \text{(S19)}
\end{aligned}$$

Therefore, $\tilde{C}_p(s)$ is written as

$$\tilde{C}_p(s) = C_p(0)\frac{\gamma_\alpha s^{\alpha-1}}{\gamma_\alpha s^\alpha + k_p} = C_p(0)\frac{s^{\alpha-1}}{s^\alpha + k_p/\gamma_\alpha}. \qquad \text{(S20)}$$

In addition, using the formula of the Laplace transform for the Mittag-Leffler function

$$\mathcal{L}\Big[E_\alpha\left(-at^\alpha\right)\Big](s) = \frac{s^{\alpha-1}}{s^\alpha + a}, \qquad \text{(S21)}$$

we can inversely find the solution

$$C_p(t) = C_p(0)\,E_\alpha\left(-k_p/\gamma_\alpha \cdot t^\alpha\right). \qquad \text{(S22)}$$

By use of Eqs. 6 and 7,

$$\frac{k_p}{\gamma_\alpha} = \frac{A_{d_{\rm f}}\cdot 3k_{\rm B}T}{N\gamma_\alpha\langle R^2\rangle_{\rm CD}}\,p^{1+2/d_{\rm f}}. \qquad \text{(S23)}$$

Then, we can define the relaxation time

$$\tau_{d_{\rm f},\alpha} \equiv \left(\frac{N\gamma_\alpha\langle R^2\rangle_{\rm CD}}{A_{d_{\rm f}}\cdot 3k_{\rm B}T}\right)^{1/\alpha}, \qquad \text{(S24)}$$

which has the physical dimension s. If the initial condition reaches thermal equilibrium, $C_p(0)$ becomes $\langle \boldsymbol{X}_p^2\rangle_{\rm CD}$. Thus, finally, we can derive the solution

$$C_p(t) = \langle \boldsymbol{X}_p^2\rangle_{\rm CD}\,E_\alpha\left[-p^{1+2/d_{\rm f}}\left(t/\tau_{d_{\rm f},\alpha}\right)^\alpha\right]. \qquad \text{(S25)}$$

E. The MSD of the center of the CD

For $p = 0$, the normal coordinate $\boldsymbol{X}_0(t)$ corresponds to the center of the CD,

$$\boldsymbol{R}_{\mathrm{G}}(t) = \frac{1}{N}\int_0^N \boldsymbol{R}(n,t)\,dn. \tag{S26}$$

According to the Langevin equation (Eq. 5) and the FDR (Eq. S2) for $p = 0$, the motion obeys

$$\int_0^t \gamma(t-t')\frac{d\boldsymbol{X}_0(t')}{dt'}\,dt' = \boldsymbol{g}_0(t), \qquad \text{where} \quad \langle g_{0\kappa}(t) g_{0\lambda}(t')\rangle = \frac{k_{\mathrm{B}}T}{N}\gamma(t-t')\delta_{\kappa\lambda}. \tag{S27}$$

In general, for degree of freedom x and velocity v, the MSD is associated with the velocity correlation as follows:

$$\begin{aligned}\left\langle [x(t)-x(0)]^2 \right\rangle &= \left\langle \left(\int_0^t v(t_1)\,dt_1 \right)^2 \right\rangle, \\ &= \int_0^t dt_1 \int_0^t dt_2\, \langle v(t_1) v(t_2)\rangle, \\ &= 2\int_0^t dt_1 \int_{t_1}^t dt_2\, \langle v(t_1) v(t_2)\rangle, \\ &= 2\int_0^t dt_1 \int_0^{t-t_1} dt'\, \langle v(t_1) v(t_1+t')\rangle. \end{aligned} \tag{S28}$$

Using the Laplace transform and the stationarity of the velocity correlation $C_v(t)$, this relation becomes more clear:

$$\begin{aligned}\mathcal{L}\left[\left\langle [x(t)-x(0)]^2 \right\rangle\right](s) &= 2\frac{1}{s^2}\mathcal{L}\left[\langle v(0)v(t)\rangle\right](s) \\ &= \frac{2}{s^2}\tilde{C}_v(s). \end{aligned} \tag{S29}$$

In terms of the fluctuation-dissipation theorem (FDT) [S2], we can derive the Laplace transform of the velocity correlation from the relationship between the average response and the FDR. The force balance between the average response of the system described by Eq. S27 and the external force $f(t)$ is written as

$$\int_0^t \gamma(t-t')\langle v(t')\rangle\,dt' = f(t), \tag{S30}$$

for one degree of freedom. The Laplace transform of this force balance equation becomes

$$\tilde{\gamma}(s)\,\langle \tilde{v}(s)\rangle = \tilde{f}(s). \tag{S31}$$

Then, the ratio of the average velocity to the force, $\langle \tilde{v}(s)\rangle / \tilde{f}(s) = 1/\tilde{\gamma}(s)$, is called the complex admittance, and the FDT of the first kind represents the relationship between the complex admittance and the velocity correlation,

$$\frac{k_{\mathrm{B}}T}{N}\frac{1}{\tilde{\gamma}(s)} = \tilde{C}_v(s), \tag{S32}$$

where the coefficient $k_{\mathrm{B}}T/N$ is caused by the FDT of the second kind for the system in Eq. S27.

Therefore, the Laplace transform of the MSD is written as

$$\begin{aligned}\mathcal{L}\left[\left\langle [x(t)-x(0)]^2 \right\rangle\right](s) &= \frac{2}{s^2}\frac{k_{\mathrm{B}}T}{N}\frac{1}{\tilde{\gamma}(s)}, \\ &= \frac{2k_{\mathrm{B}}T}{N\gamma_\alpha}\frac{1}{s^{\alpha+1}}. \end{aligned} \tag{S33}$$

By use of the formula of the inverse Laplace transform, $\mathcal{L}^{-1}[1/s^{\alpha+1}](t) = t^{\alpha}/\Gamma(1+\alpha)$, the MSD can be obtained as

$$\left\langle [x(t) - x(0)]^2 \right\rangle = \frac{2k_{\rm B}T}{\Gamma(1+\alpha)N\gamma_\alpha}\, t^{\alpha}. \tag{S34}$$

Thus, the MSD of the center of the CD is derived as

$$\begin{aligned}\left\langle [\boldsymbol{X}_0(t) - \boldsymbol{X}_0(0)]^2 \right\rangle &= 3\,\frac{2k_{\rm B}T}{\Gamma(1+\alpha)N\gamma_\alpha}\, t^{\alpha}, \\ &= \frac{2\langle R^2\rangle_{\rm CD}}{A_{d_{\rm f}}\Gamma(1+\alpha)}\left(\frac{t}{\tau_{d_{\rm f},\alpha}}\right)^{\alpha}. \end{aligned} \tag{S35}$$

F. The MSD for $t \ll t_{d_{\rm f},\alpha}$

The MSD obtained in our experiment is calculated by averaging nucleosome movements at various positions in CDs. Then, we can replace the term $\cos^2\left(\frac{p\pi n}{N}\right)$ in Eq. 8 by the average 1/2. Therefore, for $t \ll \tau_{d_{\rm f},\alpha}$, according to Eqs. 7 and 12, and the asymptotic form of the Mittag-Leffler function, $E_\alpha(-x) \simeq \exp\left[-x/\Gamma(1+\alpha)\right]$ for $x \ll 1$, the second term in the right hand side (RHS) of Eq. 8 can be expressed as

$$\mathrm{MSD}(t) \simeq 8\sum_{p=1}^{\infty}\frac{1}{2}\frac{\langle R^2\rangle_{\rm CD}}{2A_{d_{\rm f}}}\frac{1}{p^{1+2/d_{\rm f}}}\left\{1 - \exp\left[-\frac{p^{1+2/d_{\rm f}}}{\Gamma(1+\alpha)}\left(\frac{t}{\tau_{d_{\rm f},\alpha}}\right)^{\alpha}\right]\right\}. \tag{S36}$$

Converting the sum into the integral, the RHS becomes

$$\frac{2\langle R^2\rangle_{\rm CD}}{A_{d_{\rm f}}}\int_0^\infty dp\,\frac{1}{p^{1+2/d_{\rm f}}}\left[1 - e^{-(t/\tau_{d_{\rm f},\alpha})^{\alpha}/\Gamma(1+\alpha)\cdot p^{1+2/d_{\rm f}}}\right]. \tag{S37}$$

Here, let us consider the integral formula calculated as follows:

$$\begin{aligned}\int_0^\infty dx\, x^{-(1+a)}\left(1 - e^{-bx^c}\right) &= \left[\left(\frac{x^{-a}}{a}\right)\left(e^{-bx^c} - 1\right)\right]_0^\infty + \frac{bc}{a}\int_0^\infty dx\, x^{-a+c-1}e^{-bx^c}, \\ &= \frac{bc}{a}\int_0^\infty \frac{1}{bc}\left(\frac{y}{b}\right)^{1/c-1} dy\left(\frac{y}{b}\right)^{(-a+c-1)/c} e^{-y}, \\ &= \frac{b^{a/c}}{a}\int_0^\infty dy\, e^{-y}y^{(1-a/c)-1}, \\ &= \frac{b^{a/c}}{a}\Gamma(1-a/c). \end{aligned} \tag{S38}$$

Therefore, the MSD for $t \ll \tau_{d_{\rm f},\alpha}$ can be written as

$$\begin{aligned}\mathrm{MSD}(t) &\simeq \frac{2\langle R^2\rangle_{\rm CD}}{A_{d_{\rm f}}}\frac{d_{\rm f}}{2}\left[\frac{(t/\tau_{d_{\rm f},\alpha})^{\alpha}}{\Gamma(1+\alpha)}\right]^{2/(2+d_{\rm f})}\Gamma\left[d_{\rm f}/(2+d_{\rm f})\right], \\ &= \frac{2\langle R^2\rangle_{\rm CD}}{A_{d_{\rm f}}\Gamma(1+\alpha)}\Gamma(1+\alpha)\frac{d_{\rm f}}{2}\left[\Gamma(1+\alpha)\right]^{-2/(2+d_{\rm f})}\Gamma\left[d_{\rm f}/(2+d_{\rm f})\right]\left(\frac{t}{\tau_{d_{\rm f},\alpha}}\right)^{\alpha\cdot 2/(2+d_{\rm f})}, \\ &= \frac{2B_{d_{\rm f},\alpha}\langle R^2\rangle_{\rm CD}}{A_{d_{\rm f}}\Gamma(1+\alpha)}\left(\frac{t}{\tau_{d_{\rm f},\alpha}}\right)^{\alpha\cdot 2/(2+d_{\rm f})}, \end{aligned} \tag{S39}$$

where

$$B_{d_{\rm f},\alpha} = \frac{d_{\rm f}}{2}\left[\Gamma(1+\alpha)\right]^{d_{\rm f}/(2+d_{\rm f})}\Gamma\left[d_{\rm f}/(2+d_{\rm f})\right] \tag{S40}$$

is a dimensionless constant depending on $d_{\rm f}$ and α.

II. REMARKS ON THE HYDRODYNAMIC EFFECT FOR OUR POLYMER MODEL

In describing the Langevin equation of polymers with the hydrodynamic interaction, the interaction affects the mobility matrix [S1, S5]. This situation corresponds to an ideal case where hydrodynamic interactions are not screened. Calculating the effect of the mobility matrix for the normal coordinates $\boldsymbol{X}_p(t)$ under the preaveraging approximation, k_p in Eq. 5 is changed into $\bar{k}_p$ with the following p-dependence:

$$\bar{k}_p \sim k_p \cdot p^{1/d-1} \sim p^{3/d}. \tag{S41}$$

Therefore, when we calculate the MSD as above, we need to calculate the integral

$$\int_0^\infty dp \, \frac{1}{p^{1+2/d}} \left[1 - e^{-(t/\tau)^\alpha/\Gamma(1+\alpha)\cdot p^{3/d}}\right]. \tag{S42}$$

By use of the integral formula (Eq. S38), the scaling of the MSD for $t \ll \tau$ can be written as

$$\mathrm{MSD}(t) \sim t^{\alpha\cdot 2/3}. \tag{S43}$$

This means that the hydrodynamic interaction cancels out the effect of the size scaling described by the fractal dimension d_f, and that the exponent of the MSD depends on only the exponent α, which relates to the memory effect of the viscoelastic medium.

[S1] Doi M, Edwards SF. The Theory of Polymer Dynamics. Oxford University Press; 1988.

[S2] Kubo R, Toda M, Hashitsume N. Statisitical Physics II: Nonequilibrium Statisitical Mechanics. 2nd ed. Springer-Verlag, Berlin, Heidelberg; 1991.

[S3] Weber SC, Theriot JA, Spakowitz AJ. Subdiffusive motion of a polymer composed of subdiffusive monomers. Phys Rev E. 2010;82:011913. doi:10.1103/PhysRevE.82.011913.

[S4] Gardiner C. Stochastic Methods: A Handbook for the Natural and Social Sciences. 4th ed. Springer, Heidelberg; 2009.

[S5] Zimm BH. Dynamics of Polymer Molecules in Dilute Solution: Viscoelasticity, Flow Birefringence and Dielectric Loss. J Chem Phys. 1956;24(2):269–278. doi:10.1063/1.1742462.
