# Supplementary figures and images for: Dynamic Nucleosome Movement Provides Structural Information of Topological Chromatin Domains in Living Human Cells
Source: PLoS Comput Biol. 2016 Oct 20;12(10):e1005136. doi: 10.1371/journal.pcbi.1005136 (PMC5072619; doi:10.1371/journal.pcbi.1005136)

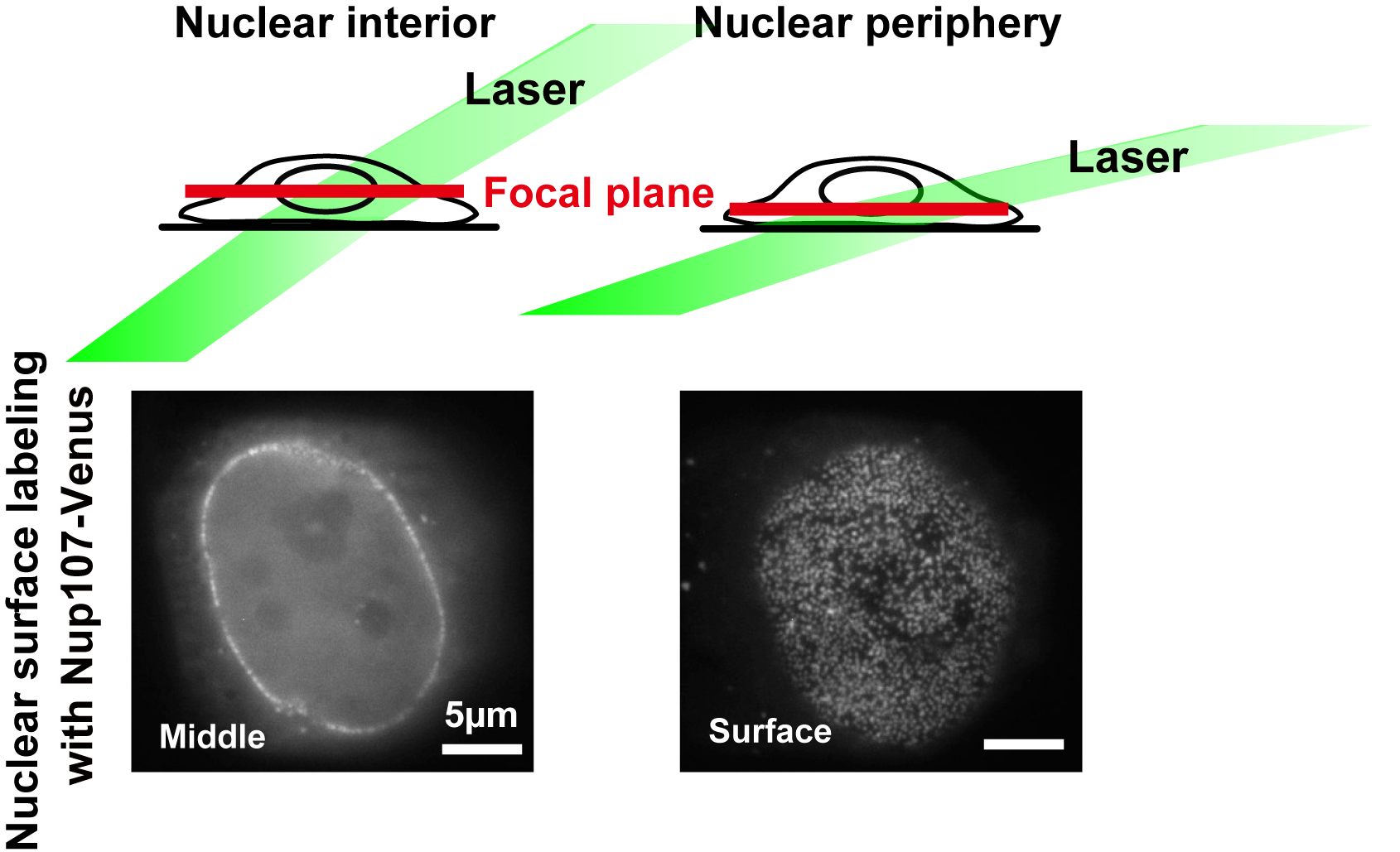

Supplement: S1 Fig — Illumination laser (green) and focal plane (red) in the living cells are shown. Note that the two different focal planes were precisely verified by nuclear surface labeling with Nup107 (a nuclear pore component)-Venus (a bright yellow fluorescent protein) [63]. The nuclear rim signals (Bottom left) and dot signals in ellipse shape (Bottom right) show the middle layer of nucleoplasm and the nuclear surface, respectively. Bar shows 5 μm. (TIF) [file pcbi.1005136.s002.tif]
