# Supplementary material for: Dynamic Nucleosome Movement Provides Structural Information of Topological Chromatin Domains in Living Human Cells
Source: PLoS Comput Biol. 2016 Oct 20;12(10):e1005136. doi: 10.1371/journal.pcbi.1005136 (PMC5072619; doi:10.1371/journal.pcbi.1005136)
Supplement: S1 Table — The measurements at each region were performed using 10 cells. (PDF) [file pcbi.1005136.s005.pdf]

S1 Table. The number of tracked trajectories M_i and the standard error of the mean (SEM) of the MSD at the nuclear interior region and the periphery region. The measurements at each region were performed using 10 cells.

	Interior		Periphery	
t_i (ms)	M_i	SEM of MSD(t_i)	M_i	SEM of MSD(t_i)
50	47797	0.0000246	35289	0.0000264
100	47732	0.0000361	35267	0.0000367
150	47498	0.0000424	35176	0.0000418
200	36291	0.0000550	26072	0.0000556
250	27876	0.0000683	19614	0.0000686
300	21625	0.0000817	14572	0.0000839
350	17074	0.0000957	11008	0.0001017
400	13650	0.0001093	8533	0.0001190
450	11047	0.0001240	6825	0.0001331
500	9047	0.0001371	5491	0.0001510
